# Multiscale Interlaminar Enhancement of CNT Network/CF Hybrid Composites and In Situ Monitoring of Crack Propagation Behavior

**DOI:** 10.3390/polym18020293

**Published:** 2026-01-21

**Authors:** Tianshu Li, Fenghui Shi, Hongchen Yan, Min Li, Shaokai Wang, Yizhuo Gu, Baoyan Zhang

**Affiliations:** 1 AVIC Manufacturing Technology Institute Composite Technology Center, Beijing 101300, China; buaalitianshu@126.com (T.L.);; 2 Key Laboratory of Aerospace Advanced Materials and Performance, Ministry of Education, School of Materials Science and Engineering, Beihang University, Beijing 100191, China

**Keywords:** carbon nanotubes network, hybrid, electrical properties, process monitoring

## Abstract

It has long been desired to achieve mechanical enhancement and structural health monitoring by introducing carbon nanotubes (CNTs) into traditional carbon fiber (CF) composites. Herein, the initiation of micro-damage and crack propagation has been investigated by utilizing in situ electrical resistance changes in interlaminar hybrid CNT network/CF composites during the shear loading process. The results show a clear relationship between the crack propagation and the electrical resistance response particularly when approaching the failure of the single-layer CNT network hybrid composites. Furthermore, the chemically modified CNT network exhibits evident enhancement on main mechanical properties of the CF composites, superior to the thermoplastic toughening method. The characterizations manifest that the multiscale interlayered CNT/CF structure can simultaneously resist the crack propagation along both the in-plane direction and the cross-plane direction, which consequently enhances the flexural and compressive strengths of the composite material. This discovery provides a novel idea for the potential application of CNT network/CF hybrid composites in the integration of mechanical reinforcement and structural health monitoring, namely, that the CNT network acts not only as a reinforcing phase but also as a sensor for the structural health monitoring of the composites.

## 1. Introduction

Tremendous advances in carbon fiber-reinforced polymer (CFRP) composites have provided strong support for automotive, marine, aerospace, and other industrial fields due to their advantages of light weight, high strength, and excellent designability [1,2,3,4]. However, the weak interlaminar performance of CFRP composites remains a major challenge for their efficient application in thin-wall structures [5,6]. Although methods such as stitching, Z-pin, and three-dimensional weaving can enhance interlaminar performance, the loss of in-plane properties of the composites is inevitable [7,8,9]. Modifications aimed at toughening the resin matrix by introducing second-phase particles such as thermoplastic particles, carbon nanotubes, and graphene to extend the crack propagation path in the resin have been extensively studied. However, the composite property improvement of this type of treatment is limited by the increased resin viscosity and the reduced processability [10,11].

Benefiting from the high strength and toughness of CNT macrostructures such as arrays and films [12,13,14,15], CF/CNT hybrid composites prepared by introducing CNT macrostructures into the interlayers of CFRPs for interlaminar enhancement have achieved promising results. Wardle’s research group conducted numerous studies on the interlaminar enhancement of CFRP by using CNT arrays and proposed the concept of “nano-stitching” [16,17,18,19,20]. Many studies have indicated that CNT macrostructures, thickness (height), etc., affected the interlaminar enhancement effect. Falzon et al. transferred the vertically aligned CNT (VACNT) arrays to the prepreg and fabricated a CNT/CF hybrid composite [21]. The Mode I and Mode II interlaminar fracture toughness of the hybrid composite were increased by 61% and 161%, respectively. Song et al. found that the introduction of 100 µm VACNT arrays had no significant effect on the interlaminar shear strength (ILSS) of CF/epoxy composites, while 200 µm VACNT arrays increased the ILSS by 80% [22]. Besides the interlaminar properties, the CNT macrostructures also affected the in-plane mechanical properties of composites. The CNT arrays improved the tension-bearing critical strength, open-hole compression ultimate strength, and L-section bending energy of the composite laminate [23]. The structure and content of different CNT films also changed the tensile properties [24,25] and bending properties [26] of composite materials, and too high CNT content even significantly reduced the in-plane mechanical properties [24,26]. Specifically, after the CNT content of the hybrid composites was increased from 4.95% to 7.99%, the tensile strength decreased from 2519 MPa to 1966 Mpa [24]. In addition, when the content of hybrid CNTs was increased from 0.22% to 1.09%, the flexural strength and interlaminar shear strength (ILSS) showed an opposite trend of reduction instead [26]. These studies have focused on the effects of CNT structure and content on the interlaminar and in-plane mechanical properties. However, as a newly introduced interlaminar reinforcement, the CNT network structure with a CNT/resin interface will significantly alter the initiation and propagation of cracks in the interlayers of the composites under external forces, thereby leading to differences in mechanical properties and failure modes. These phenomena have not been focused on yet.

In addition, the excellent conductivity of the CNT network endows it with promising application prospects in stress and structural monitoring, which provides the possibility for the integrated application of mechanical enhancement and structural monitoring and is expected to ensure the structural safety of the composites during service [27,28,29,30,31,32,33]. In most structural monitoring studies, in order to avoid the influence of conductive CF on electrical signals, glass fiber-reinforced polymer (GFRP) composites are used. Gao et al. dispersed CNTs in epoxy/plain woven GFRP and discovered that the electrical resistance of the composites increased with repeated impact loading [34]. Alexopoulos et al. embedded CNT fibers to GFRP and established the relationship between tensile or three-point bending loads and the electrical resistance changes of CNT fibers [35]. Wang et al. examined the dynamic stability and durability of the CNT buckypaper sensor under monotonic and cyclic flexural loading [36]. Shen et al. introduced CNT buckypapers into GFRP with different lay-up angles and analyzed their electrical resistance response under tensile load [37]. However, high-performance CF is the most widely used reinforcement in the aerospace field compared to glass fiber, so the structural monitoring of CFRPs has better research value, but negligible research was involved [38]. At the same time, CNT materials were mostly used as sensors embedded in composites alone or arranged on the surface of composites, which could not exert their structure-enhancing effect in these studies. Analyzing the correlation among load response, crack propagation, and electrical signal changes induced by CNT reinforcements in CFRPs and utilizing them for structural monitoring are expected to promote the integrated structure–function application of CNT/CF hybrid composites.

In order to take advantage of the excellent mechanical enhancement and electrical resistance response characteristics of the CNT network at the same time and to reveal the potential synergistic mechanism, pristine and chemically modified CNT networks were used to prepare CNT/CF hybrid composites. The interlaminar enhancement mechanism of the CNT network was analyzed in comparison with a typical thermoplastic interlaminar toughened CF composite. With the help of in situ crack observation equipment and online electrical resistance monitoring, the respondence of the damage propagation and resistance change with the external loading was investigated during the interlaminar shear test of CNT/CF hybrid composites. Similar experiments were also conducted during the flexural tests. The influence of the CNT network structure and the CNT/resin interface was explored. The results provided a novel reference for potential applications of mechanical reinforcement integrated with structure monitoring of CNT/CF hybrid composites.

## 2. Experiment and Characterization

### 2.1. Materials

A unidirectional aerospace-grade T700/bismaleimide (BMI) prepreg was supplied by AVIC Composites Co., Ltd., Beijing, China and had a resin content of 32 ± 2 wt%, a volatile content of less than 0.5 wt% and an areal density of 193 ± 4 g/m^2^. The thickness of the single-layer was approximately 0.125 mm after curing. A randomly oriented CNT film was synthesized by the floating catalytic chemical vapor deposition (FCCVD) method, supplied by Jiedi Nano Technology Co., Ltd., Suzhou, China. Pristine CNT films with a thickness of 2–4 μm and CNTs of 3–7 walls and an average diameter of 6–10 nm were utilized. Herein, the pristine CNT films are denoted as CNT. To achieve stronger interfacial bonding between CNT and resin, CNT films were treated with m-chloroperoxybenzoic acid. This treatment enables the grafting of epoxy groups onto the surface of CNTs, which can chemically react with the resin. The CNT films modified via this approach were denoted as ECNT. For detailed chemical treatments of ECNT, please refer to our previous studies [39].

### 2.2. Preparation of Hybrid Composites

The thermoplastic-toughened T700/BMI prepreg was laid in the 0° direction, and the winding direction of the CNT network was the same as the 0° direction of the CF prepreg. The CNT films were placed on the prepreg layer with different stacking sequences. The abbreviations of diverse composite samples are shown in Table S1, wherein the subscripts CF, CNT, and ECNT represent the CF prepreg, the pristine CNT network, and the chemically treated CNT network, respectively. The abbreviations TTCFC, CNTHC, and ECNTHC denote the thermoplastic-toughened CF composite, the CNT network hybrid composite, and the ECNT network hybrid composite, respectively. The subscripts 1/4, 1/2, and 3/4 accordingly indicate that the single-layer CNT network is located at the 1/4, 1/2, and 3/4 positions from the top to the bottom of the composite. All composites were cured in an autoclave following the manufacturer’s specifications: 0.6 MPa of total pressure at 1 °C/min to 150 °C, hold for 1 h, heat again at 1 °C/min to 180 °C, hold for 3 h, heat again at 1 °C/min to 200 °C, and hold for 5 h. Then cool down at 1.5 °C/min to room temperature and vented pressure.

### 2.3. Characterization Methods

The mechanical properties of the composites were all tested using an Instron Corporation’s Instron 3382 universal testing machine, Norwood, MA, USA. The interlaminar shear strength was tested using a short-beam method according to ISO 14130:1997 [40]. The longitudinal compression and flexural properties were tested according to ASTM D6641-16 [41] and ASTM D7264-15 [42] standards, respectively. The nanoindentation modulus was tested using an Anton Paar GmbH’s Anton Paar STEP500 nanoindentation instrument, Graz, Austria, for which a triangular indenter, a pressing load of 2–5 mN, and a pressing spacing of 2–3 μm were selected.

**Figure 1:**
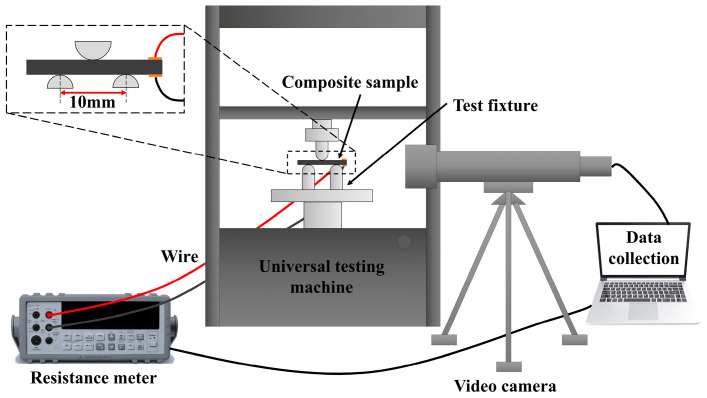
Schematic diagram of in situ crack observation and electrical resistance measurement during the interlaminar shear test.

The in situ crack observation and online electrical resistance measurement were conducted during the mechanical test process, as shown in Figure 1. The in situ crack observation was realized by a Jike Instrument Co., Ltd.’s JK 20TM002 observation instrument, Beijing, China, with a 0.2 s/time shooting speed and a 50× magnification. The inserted illustration in Figure 1 showed the span of the interlaminar shear test and the connections of the resistance test. The electrical resistance signals along the cross-plane direction of the samples were collected every 0.1 s with an accuracy of 0.01 mΩ using a Keithley Instruments, Inc.’s Keithley DAQ 6510 multimeter system, Solon, OH, USA. The resistance of each specimen was measured 5 times at the same position to determine the resistance measurement error. Through calculation, the measurement error of the resistance meter was determined to be 0.05%.

Macroscopic fracture morphologies of the specimens were obtained by the Keyence Corporation’s Keyence VHX-6000 3D digital microscope, Osaka, Japan. By utilizing the 3D depth synthesis function, images of the compression fracture were obtained. Further microscopic fracture morphologies of the specimens were observed by a JEOL Ltd.’s JSM 7500F field emission scanning electron microscope (FESEM), Tokyo, Japan, with a 3 kV acceleration voltage at a 7~9 mm working distance. The damages of the specimens were also characterized by the ultrasonic C-scan method using the Evident Scientific’s Olympus Omniscan SX ultrasonic scanning system, Tokyo, Japan.

## 3. Results and Discussion

### 3.1. Multiscale Interlaminar Enhancement of CNT/CF Hybrid Composites

**Figure 2:**
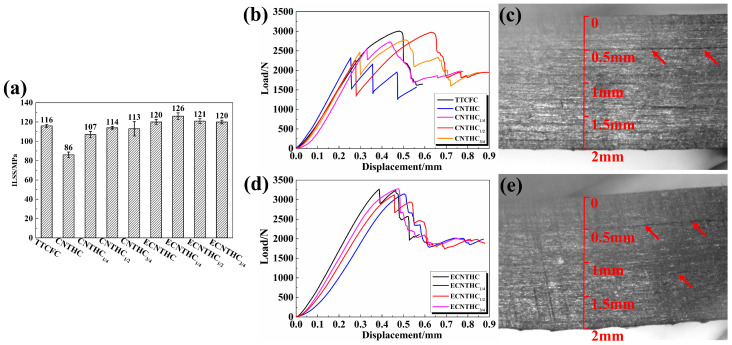
Interlaminar shear strength of (**a**) CNT network hybrid composites and ECNT network hybrid composites; interlaminar shear load–displacement curves of (**b**) CNT network hybrid composites and (**d**) ECNT network hybrid composites; in situ first crack propagation in (**c**) CNTHC_1/4_ and (**e**) ECNTHC_1/4_.

Firstly, the effects of the pristine CNT network and the chemically modified ECNT network on the interlaminar properties were analyzed and compared with those of a commercially available thermoplastic-toughened composite. As shown in Figure 2a, the ILSSs of CNTHC_1/4_, CNTHC_1/2_, and CNTHC_3/4_ ranged from 107 to 113 MPa, which were significantly higher than those of CNTHC (86 MPa). The ILSSs of all the single-layer CNT network hybrid composites were comparable to that of TTCFC (116 MPa). In Figure 2b, the load of CNTHC decreased firstly at 2300 N. As a contrast, the first load drop of CNTHC_1/4_, CNTHC_1/2_, and CNTHC_3/4_ occurred at around 2500 N, then further rose to about 3000 N with a second delamination. Figure 2c shows that the first crack of CNTHC_1/4_ appeared at the 0.5 mm position of the laminate, revealing the first shear crack generated within the CNT layer. Other CNT network hybrid composites experienced the same situation as shown in Figure S2, which indicated weak interactions among the CNT sublayers. Owing to the high ductility of the CNT films, the hybrid composites could continue to withstand higher loads until delamination occurred within the CF layers. Thus, the single-layer CNT network in CNTHC_1/4_, CNTHC_1/2_, and CNTHC_3/4_ hardly affected the ILSSs of the composites while the multi-layer CNT networks in CNTHC greatly reduced its ILSS.

In Figure 2a, the ILSSs of ECNTHC, ECNTHC_1/2_, and ECNTHC_3/4_ were 120~121 MPa, whereas that of ECNTHC_1/4_ was 126 MPa. Comparison with the ILSS of TTCFC demonstrated that the ECNT network effectively increased the ILSSs of the carbon fiber composites. Figure 2d shows the similar interlaminar shear load–displacement curves of these hybrid composite samples, all of which exhibited peak loads of more than 3000 N as the first delamination occurred. As shown in Figure 2e, the first load drop of the ECNTHC_1/4_ was accompanied by several local cracks, located within the ECNT layer and the CF layers, respectively. The simultaneous occurrence of different cracks indicated that the resistance of the ECNT network to interlaminar shear load was comparable to that of the thermoplastic particle-toughened sublayers. Hence, the ECNT network effectively enhanced the ILSSs of the hybrid composites.

**Figure 3:**
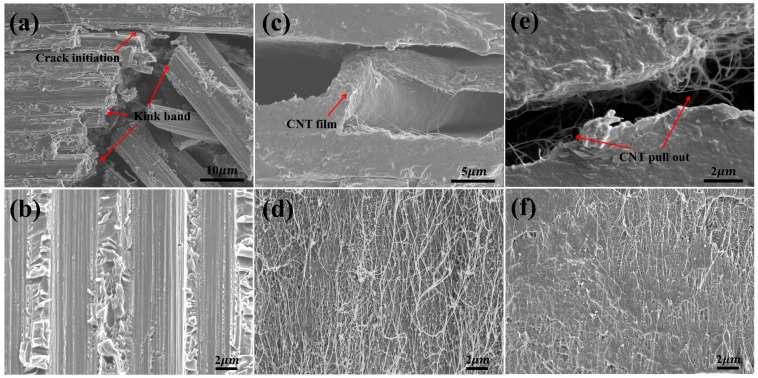
Lateral interlaminar shear failure morphologies of (**a**) TTCFC, (**c**) CNTHC, and (**e**) ECNTHC; internal interlaminar shear failure morphologies of (**b**) TTCFC, (**d**) CNTHC, and (**f**) ECNTHC.

The lateral and internal morphologies of the shear fractures were characterized by SEM to analyze the effects of the CNT network structure and the nanoscale CNT/resin interface on the ILSSs of the hybrid composites, as shown in Figure 3. For the thermoplastic-toughened TTCFC, kink band failure first appeared inside the CF layers, as shown in Figure 3a. This was attributed to the considerably lower mechanical properties and poor stiffness of the thermoplastic-toughened layers, which could not provide rigid support for the CF layers. Then, at the end of the kink band, interlayer cracks propagated along the in-plane direction. As shown in Figure 3b, the resin between adjacent CF layers displayed a shear lip morphology, demonstrating toughness shear failure.

For CNTHC, Figure 3c shows that delamination failure occurred at both sides of the CNT network, showing smooth fracture surfaces, which was indicative of poor interactions between the CF layer and the CNT film. As shown in Figure 3d, no resin adhesion could be seen on the fractured CNT network surface. This proved that the pristine CNTs had a weak nanoscale CNT/resin interface, due to their inert surfaces and even small organic molecule byproducts on the surface of the pristine CNTs. Both induced an ILSS of 86 MPa, much lower than that of TTCFC (116 MPa). Hence, under relatively small shear stress, the nanoscale CNT/resin interface debonded at first. Then cracks rapidly propagated along the two-dimensional CNT network structure, causing delamination failure of the entire interlayer of CNTHC.

As for ECNTHC, a large number of CNTs were pulled out at the two adjacent fracture surfaces in Figure 3e, suggesting apparent delamination failure between the sublayers in the CNT film. In Figure 3f, the remnant resin covered most nano and micron structures on the fractured ECNT network surface. Only a small amount of CNT pull-outs could be identified, demonstrating strong nanoscale interfacial strength between the BMI resin and ECNTs due to the significantly increased active functional groups on ECNTs after chemical treatment. As reported, the interfacial shear strength (IFSS) of m-CPBA-treated CNT/resin can be increased by one order of magnitude [43]. Thereby, the ECNT multiscale interlayer exhibited strong resistance to shear load, and the ILSS of the ECNTHC hybrid composite was significantly improved to more than 120 MPa.

### 3.2. In Situ Resistance Monitoring of Crack Propagation in CNT/CF Hybrid Composites

**Figure 4:**
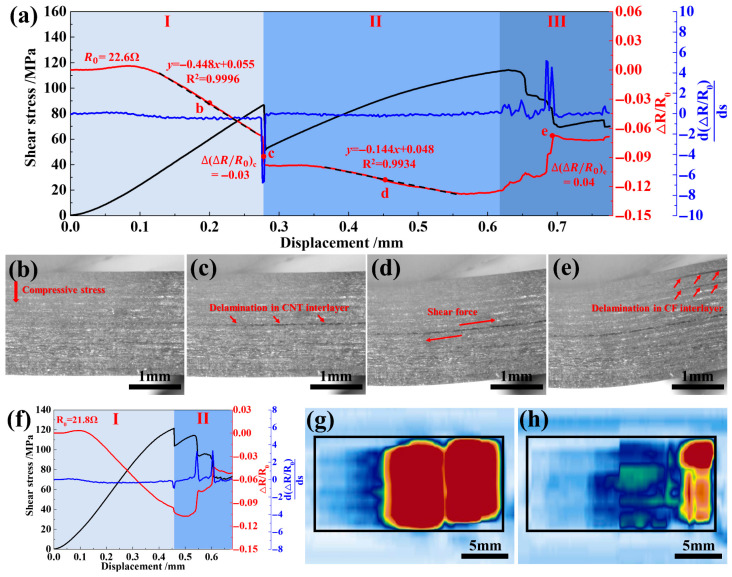
Interlayer shear displacement, load, and $∆R/R_{0}$, $\frac{d(∆R/R_{0})}{ds}$ curves of (**a**) CNTHC_1/2_ and (**f**) ECNTHC_1/2_ composites; in situ crack propagation of CNTHC_1/2_ at (**b**) point b, (**c**) point c, (**d**) point d, and (**e**) point e; interlayer delamination of (**g**) CNTHC_1/2_ and (**h**) ECNTHC_1/4_ composites by ultrasonic C scan.

The CNT network has excellent electrical conductivity. When cracks propagated in the CNT network layers, the conductive paths were cut off, which inevitably would affect the electrical resistance of the composite. Hence, monitoring the electrical resistance changes under interlaminar shear loads was expected to determine the warning state of shear stress and the structural delamination inside the composites. Herein, the variation trends of the external load, the through-thickness electrical resistance change ($∆R/R_{0}$), and the rate of change of electrical resistance ($\frac{d(∆R/R_{0})}{ds}$) of CNTHC_1/2_ were investigated as a function of displacement (*s*). The results are shown in Figure 4a. Initially, the $∆R/R_{0}$ curve of CNTHC_1/2_ was almost flat with increasing displacement, when the stress distribution inside the entire composite was uneven. Then the electrical resistance displayed a linearly decreasing tendency at the latter part of stage I in Figure 4a. Correspondingly, the spaces between CFs were reduced uniformly under compressive stress, as shown in Figure 4b. With the shear stress further increased, a sudden drop in stress took place with simultaneous occurrence of delamination inside the CNT film, as shown in Figure 4c. Accordingly, the through-thickness electrical resistance change $∆R/R_{0}$ of the composite exhibited a sudden drop of 3%, and a dramatic decrease could also be observed from the $\frac{d(∆R/R_{0})}{ds}$ curve. Hence, the electrical resistance of the hybrid composite had an obvious and sensitive response to the occurrence of delamination in the CNT sublayers. When the shear stress in the composite reached the pre-warning level, the failure of the weak nanoscale CNT/resin interface induced crack propagation, leading to an obvious signal feedback of electrical resistance, which can be utilized to realize the early warning of the composite structure health state.

After the occurrence of a delamination in CNT film, the hybrid composite CNTHC_1/2_ continued to withstand higher shear load. Likewise, the $∆R/R_{0}$ curve remained almost changeless and then decreased linearly with the increasing displacement in stage II. The former tendency should be ascribed to stress redistribution after the delamination took place in the composite. The latter declination of $∆R/R_{0}$ was slightly small than that in stage I, which should be caused by release of the compressive stress due to the shear slip near the interlayer cracks, as shown in Figure 4d. With a further increase in the shear load, the deflection of the composite CNTHC_1/2_ increased and delamination cracks also occurred in the CF layers near the sample end, as shown in Figure 4e. The destroyed conductive paths between the CF layers and in the CNT film both resulted a continuous increase in $∆R/R_{0}$ in stage III. Thereby, the relationship between $∆R/R_{0}$ and displacement corresponded to the evolution of internal damages of the hybrid composite as well as the stress state during the interlaminar shear test process. According to the in situ electrical resistance curve, the initiation of cracks could be monitored by the mutation of the $∆R/R_{0}$, and the final multiple delamination within the composite could also be monitored by the step-increase tendency of $∆R/R_{0}$ for the last stage.

In comparison, the performance of ECNTHC_1/2_ was analyzed at the same time. In Figure 4f, the $∆R/R_{0}$ presented an unchanged firstly and a subsequent decline tendency, similar with that of CNTHC in stage I. However, the decrement in $∆R/R_{0}$ of ECNTHC_1/2_ was only 0.5%, which was much smaller than the 3% decline of CNTHC_1/2_ due to the occurrence of delamination within the CNT layer in stage I. Herein, the shear load borne by ECNTHC_1/2_ kept increasing until it reached the maximum point at the end of stage I. Then the electrical resistance changed frequently due to the emergence of diverse delamination inside composite ECNTHC_1/2_, which agreed well with the decrease in the shear stress in stage II. The final damages of the two types of hybrid composites were characterized by an ultrasonic C scan after the interlaminar shear tests. In Figure 4g, a serious delamination with a size of 111.1 mm^2^ was identified in CNTHC_1/2_, displayed as a red color zone, whereas in Figure 4h it was only 27.0 mm^2^ for ECNTHC_1/2_. Some studies have shown that the electrical resistance change of the composite is related not only to the delamination area but also to the conductive paths formed by the dense stacking of the CNT network and the nanoscale CNT/resin interface in the multiscale interlaminar hybrid structure [44,45]. Therefore, the following equation was established to analyze the effects of various factors, including the area of the serious delamination area in the composite (*S*) and the electrical resistivity of the CNT network (*ρ*), on the change of $∆R/R_{0}$:

a=η·S·ρ

where *a* was the absolute value of the change ratio of $∆R/R_{0}$ at the sudden load change, representing the sensitivity of the electrical resistance response when delamination occurs. *η* was defined as the correction factor related to the sparse CNT network structure and the weak CNT/resin interface in the multiscale interlayer structure. In Table 1, the values of *a* are given in Figure 4a and f, respectively. The values of *S* were obtained by ultrasonic C-scan, as shown in Figure 4g,h. The values of *ρ* were obtained by the electrical resistivity tests of the CNT network and ECNT network. Then *η* could be calculated.

The *ρ* and *η* values of CNTHC_1/2_ were higher than those of ECNTHC_1/2_, which was more conducive to a larger change in *a*. However, the value of *S* showed a dominant effect on the value of *a*. Although more delamination was the main factor to improve the sensitivity of electrical resistance change, the safety of composite structures was also adversely affected. Therefore, subsequent research should focus on improving the sensitivity, e.g., by increasing the electrical resistivity of the conductive network and reducing the density of the CNT network in the multiscale interlayered structure. This is because a higher resistivity will lead to a more significant resistance change per unit damage area, thereby facilitating easier detection. In conclusion, the construction of conductive networks with significant electrical resistance changes when delamination occurs in the interlayers of the hybrid composite is the key to realizing shear stress warning and structure health state monitoring.

In addition, studies in the structure monitoring field have mostly selected GFRP or set an insulating layer outside the conductive sensor to eliminate the effect of the conductivity of the CF [34,37,44,46], which has limited its application prospects. The signal detected in this study was the electrical resistance along the cross-plane direction of the composite. In this direction, the CF layers and the CNT layers were connected in series, and the conductivity of CFs had an insignificant contribution to the whole electrical resistance change of the hybrid composite. Eliminating the influence of the conductivity of the CF is one of the main directions to extend the application of the conductive sensor in CF composites.

### 3.3. Flexural Properties and Failure Morphologies of CNT/CF Hybrid Composites

**Figure 5:**
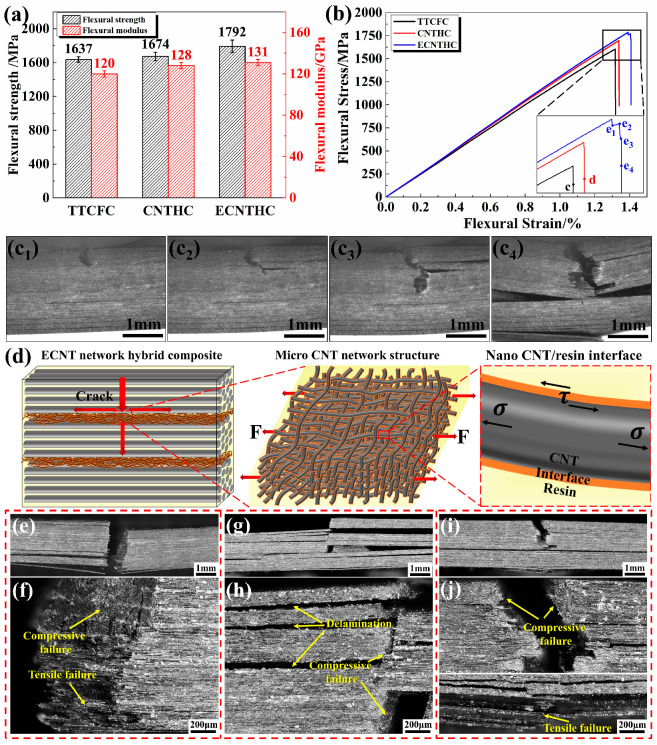
(**a**) Flexural properties and (**b**) stress–strain curves of TTCFC, CNTHC, and ECNTHC laminates. (**c_1_**–**c_4_**) In situ crack propagation images of ECNTHC laminates. (**d**) Schematic diagram of the synergistic enhancement of the multiscale interlayer structure of ECNTHC. Macroscopic and partial enlarged failure morphologies of (**e**,**f**) TTCFC, (**g**,**h**) CNTHC, and (**i**,**j**) ECNTHC after flexural tests.

To further investigate the mechanical enhancement effect of the CNT network, three-point bending tests were conducted on the CNT network/CF hybrid composites, and the performance of the thermoplastic-toughened composite (TTCFC) was analyzed as a control. For ECNTHC, the flexural strength and modulus were 1792 MPa and 131 GPa, respectively, i.e., 9.5% and 9.2% higher than those of TTCFC, as shown in Figure 5a. The flexural stress–strain curve in Figure 5b shows that the stress of TTCFC and CNTHC decreased rapidly after the highest point. The curve of ECNTHC fluctuated after the highest stress level, and a multiple-stage descending pattern was present. The bending behavior was investigated via in situ observation of crack propagation. At first, a crack initiated at the indenter location due to stress concentration (Figure 5c_1_). Then, the crack split into two and propagated along the thickness and the interlayer direction simultaneously (Figure 5c_2_). Furthermore, the cracks extended sequentially along the interlayer and the cross-plane direction (Figure 5c_3_). At last, ECNTHC fractured due to simultaneous compression and tension stresses (Figure 5c_4_). The schematic diagram of the multiscale interlaminar structure of ECNTHC is illustrated in Figure 5d, including the microscale CNT network and the nano-scale CNT/resin interface within the ECNT sublayers. During the flexural test process, both tensile and shear stresses were transmitted through the multiscale ECNT networks. The strong interface formed by chemically modified nanotubes and resin can bear shear stress (with an ILSS of 120 MPa). Crack propagation along the interlaminar direction requires breaking the high-strength ECNT/resin interface, which demands higher energy compared with that of conventional composites. Thus, ECNTs can delay crack growth along the in-plane direction. Moreover, the tensile strength of the ECNT network combined with resin can exceed 500 MPa (Figure S7c), which is much higher than the tensile strength of BMI resin (approximately 100 MPa). Therefore, the high-performance ECNT network can withstand higher tensile stress without damage even under relatively high loads, thereby delaying crack propagation along the cross-plane direction.

Typical flexural fracture morphologies of the three different composites were characterized. In Figure 5e, the macroscopic image shows that the TTCFC laminate was directly fractured into two parts with brittle fracture surfaces (Figure 5f), which is consistent with the in situ observation in Figure S4a. For the CNTHC laminate, Figure 5g shows that the upper compressive region was fractured with numerous delamination fragments, while the tensile region remained unbroken. The high-magnification image in Figure 5h shows that the delamination tended to take place adjacent to the CNT interlayers, which should be ascribed to the weak interface inside the nanotube networks. In CNTHC, the pristine nanotubes possessed weak interactions among the CNT network sublayers as well as a weak interface of nanotube/resin, which might induce crack propagation from along the cross-plane direction into the in-plane direction.

The ECNTHC laminate showed obvious damages in the compressive region but slight ones in the tensile region in Figure 5i. From the high-magnification image in Figure 5j, fewer interlaminar cracks could be observed in the upper compressive region, indicating that a strong ECNT/resin interface could hinder crack propagation along the in-plane direction but promote it in the cross-plane direction. Meanwhile, obvious CF breakage and CF pull-out could be seen in the bottom tensile region of the ECNTHC laminate in Figure 5j. In summary, both types of CNT film hybrid composites revealed more complex mixed failure modes compared to TTCFC.

### 3.4. Compressive Properties of CNT/CF Hybrid Composites

**Figure 6:**
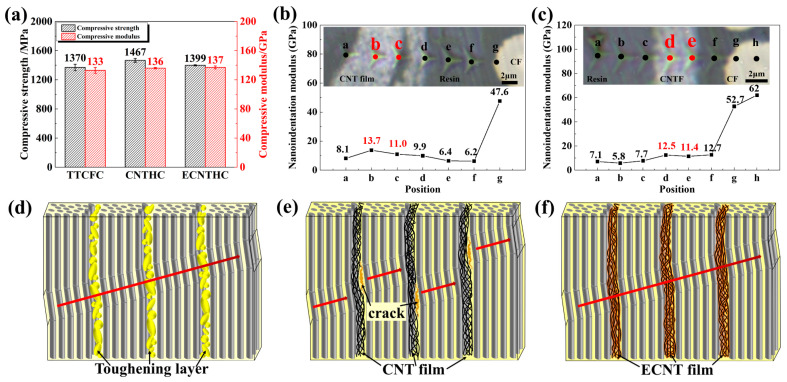
Compressive properties of different composites (**a**), nanoindentation modulus of (**b**) CNTHC and (**c**) ECNTHC, and schematic illustrations of kink band extension modes of (**d**) TTCFC, (**e**) CNTHC, and (**f**) ECNTHC.

The improvement in the longitudinal compressive performance of the hybrid composites induced by the CNT network was investigated. In Figure 6a, the compressive strengths of TTCFC and ECNTHC were 1370 MPa and 1399 MPa, respectively, whereas that of CNTHC was relatively higher, reaching 1467 MPa. The typical compressive failure morphologies of the three types of composites are shown in Figure S5. The morphologies of TTCFC and ECNTHC both showed dominant kink band fracture (Figure S5a,c), while the morphology of CNTHC exhibited numerous delamination fragments (Figure S5b). This should be attributed to the rigid support of the CNT network to the CF in the hybrid composites. Nanoindentation tests were performed on the cross-sections of CNTHC and ECNTHC. Figure 6b,c show that the CNT and ECNT networks possess nanoindentation moduli in the range of 11–14 GPa, which were much higher than the modulus values of the BMI resin and the thermoplastic particle regions (6–8 GPa). Benefiting from the stiffness-enhancing effect of the CNT network sublayers, the generation of CF micro-buckling was considerably delayed during the compression process of the hybrid composites [47,48,49]. Owing to the high interlaminar strength provided by thermoplastic toughening or the strong ECNT/resin interface, all the CF layers could synergistically withstand external compressive loads, resulting in the dominant kink band failure of TTCFC and ECNTHC, as illustrated in Figure 6d,f. In the first stage, with increasing compressive stress, a few fibers began to break off due to buckling instability. Then, the kink band appeared which expanded across the adjacent layers continuously along the direction indicated by the arrows in Figure 6d,f until the final failure of the composites. In comparison, CNTHC revealed predominant interlaminar cracks and delamination fracture, as illustrated by Figure 6e. Previous investigation manifested that the shear stress generated during the compression process had an important impact on the generation and further expansion of the kink bands [50,51]. In CNTHC, the numerous interlaminar delamination fragments could absorb a significant amount of energy due to weak interactions between the CNT network sublayers and the weak interface of CNT/resin, which released shear stress among laminae. Meanwhile, the interlayer cracks deflected the direction of the kink bands. Thanks to the stiffness-augmenting effect of the CNT layer, the occurrence of kink band failure in the CF layers was deferred. Hence, the compressive strength of the hybrid composite CNTHC was improved significantly.

## 4. Conclusions

A multiscale interlaminar structure, including a nanoscale CNT/resin interface and a microscale CNT network structure, was formed by incorporating continuous self-supported CNT films fabricated via FCCVD into the interlayers of the composites, which significantly altered the mechanical behavior and electrical resistance response of the hybrid composites. Owing to chemical modification, the strong ECNT/resin interface significantly improved the delamination resistance of the hybrid composites under shear loads and increased the ILSS to over 120 MPa. A good correlation was observed between the shear load, crack propagation, and electrical resistance changes of CNTHC during the interlaminar shear process. This can be utilized to achieve stress warning and structural state monitoring in the future. The change ratio of $∆R/R_{0}$ at the first occurrence of delamination was significantly affected by the size of the crack area. Increasing the electrical resistivity of the conductive network and reducing the density of the CNT network are the crucial points to further improving the monitoring sensitivity of the hybrid composites. In situ observation experiments demonstrated that under bending loads, the macro/micro/nano interlaminar structure can synergistically resist crack propagation both along the interlaminar direction and the cross-plane direction. Thus, the flexural strength and modulus of ECNTHC were increased by approximately 9% by changing the failure mode to a mixed mode. Under compressive loads, the CNT network acts as a stiffness-enhancing factor that provides strong support for the CF layers, delaying the occurrence of their buckling instability in the hybrid composites. Owing to the weak interactions between the CNT network sublayers and the weak CNT/resin interface, the fracture morphologies of CNTHC exhibited numerous interlaminar delamination fragments, which could relieve the shear stress among the laminae during the compression process. Therefore, CNTHC showed the highest values of compressive strength. By utilizing the mechanical enhancement effect and the electrical resistance response monitoring characteristics of the CNT network, a potential application of mechanical reinforcement integrated with structure monitoring of CNT/CF hybrid composites is proposed. This application is conducive to addressing the problems of mechanical property degradation and defect formation in composites caused by embedding independent structural health monitoring sensors.

## Figures and Tables

**Table 1:** Comparison of related parameters of $∆R/R_{0}$ in CNTHC_1/2_ and ECNTHC_1/2_.

Sample	a/%	S/mm^2^	ρ /Ω·m	η $/\Omega^{-1}\cdot m^{-3}$
CNTHC_1/2_	3.0	111.1	1.97 × 10^−5^	1.37 × 10^7^
ECNTHC_1/2_	0.5	27.0	1.63 × 10^−5^	1.14 × 10^7^

## Data Availability

The data supporting the conclusions of this study are not publicly available due to privacy restrictions, but are available from the corresponding author upon reasonable request.

## Supplementary Materials

The following supporting information can be downloaded at: https://www.mdpi.com/article/10.3390/polym18020293/s1, Figure S1: Interlaminar shear load-displacement curves of TTCFC, CNTHC and ECNTHC. In-situ crack propagation images of TTCFC, CNTHC and ECNTHC; Figure S2: First in-situ crack propagation images of the CNTHC 1/2, CNTHC 3/4, ECNTHC 1/2 and ECNTHC 3/4; Figure S3: Interlayer shear displacement-load-resistance change-resistance change derivative curve of TTCFC, interlayer delamination of TTCFC by ultrasonic C scan; Figure S4: In situ crack propagation images of TTCFC and CNTHC laminates; Figure S5: The Macroscopic 3D compressive fracture morphologies of TTCFC, CNTHC and ECNTHC which obtained by 3D digital microscope; Figure S6: Top view of the compressive fracture morphologies of TTCFC, CNTHC and ECNTHC. Local 3D fracture morphologies of TTCFC, CNTHC and ECNTHC. Contour curves in the width direction and thickness direction of the three composites; Figure S7: SEM image of the pristine CNT network before chemical treatment, SEM image of the CNT network after chemical treatment and tensile properties of the pristine CNT film and its composite; Table S1: Abbreviations and stacking sequences of every composites. Reference [52] is cited in Supplementary Materials.
