# Supplementary material for: Multiscale Interlaminar Enhancement of CNT Network/CF Hybrid Composites and In Situ Monitoring of Crack Propagation Behavior
Source: Polymers (Basel). 2026 Jan 21;18(2):293. doi: 10.3390/polym18020293 (PMC12845759; doi:10.3390/polym18020293)
Supplement: Supplementary file 1 [file polymers-18-00293-s001.zip › polymers-4095256-supplementary.pdf]

Supporting information

Multiscale Interlaminar Enhancement of CNT Network/CF Hybrid Composites and In Situ Monitoring of Crack Propagation Behavior

Tianshu Li [1], Fenghui Shi [1,*], Hongchen Yan [1], Min Li [2,*], Shaokai Wang [2], Yizhuo Gu [2] and Baoyan Zhang [1]

[1] AVIC Manufacturing Technology Institute Composite Technology Center, Beijing 101300, China
[2] Key Laboratory of Aerospace Advanced Materials and Performance, Ministry of Education, School of Materials Science and Engineering, Beihang University, Beijing 100191, China
* Correspondence: fenghuishi@126.com (F.S.); leemy@buaa.edu.cn (M.L.)

S1. Preparation of hybrid composites

The randomly oriented CNT film was synthesized by FCCVD method. In this method, a mixed liquid of ethanol (carbon source), ferrocene (2.0 wt%, catalyst precursor) and thiophene (1 wt%, promoter) was injected into a reaction furnace (1300ºC) at a rate of 0.15 mL/min. An Ar-H_2 mixture (volume ratio of 1: 1) was also injected as a carrier gas.

Table S1 showed the abbreviations and specific stacking sequences of every composites.

Table S1. Abbreviations and stacking sequences of every composites

Abbreviation	Stacking sequences	Specific stacking sequences
TTCFC	$[0_{CF}]_{16}$	Sixteen CF layers were stacked in 0°direction.
CNTHC	$[0_{CF}/\overline{0_{CNT}}]_{8S}$	The CF layer and CNT network were alternately stacked, in which the intermediate plane of symmetry was the thin network.
ECNTHC	$[0_{CF}/\overline{0_{ECNT}}]_{8S}$	The CF layer and ECNT network were alternately stacked, in which the intermediate plane of symmetry was the thin network.
CNTHC $_{1/4}$	$[0_{CF4}/0_{CNT}/0_{CF12}]$	Sixteen CF layers were stacked in 0°direction and a CNT network was stacked between fourth and fifth CF layers.
CNTHC $_{1/2}$	$[0_{CF8}/\overline{0_{CNT}}]_{S}$	Sixteen CF layers were stacked in 0°direction and a CNT network was stacked between eighth and ninth CF layers.
CNTHC $_{3/4}$	$[0_{CF12}/0_{CNT}/0_{CF4}]$	Sixteen CF layers were stacked in 0°direction and a CNT network was stacked between twelfth and thirteenth CF layers.
ECNTHC $_{1/4}$	$[0_{CF4}/0_{ECNT}/0_{CF12}]$	Sixteen CF layers were stacked in 0°direction and a ECNT

		network was stacked between fourth and fifth CF layers.
ECNTHC $_{1/2}$	$[0_{CF8}/\overline{0_{ECNT}}]_S$	Sixteen CF layers were stacked in 0°direction and a ECNT network was stacked between eighth and ninth CF layers.
ECNTHC $_{3/4}$	$[0_{CF12}/0_{ECNT}/0_{CF4}]$	Sixteen CF layers were stacked in 0°direction and a ECNT network was stacked between twelfth and thirteenth CF layers.

S2. Results and discussion

According to the load-displacement curve shown in Fig. S1(a) and the in-situ crack propagation images of Fig. S1 (b)~(d), the effect of different CNT networks on the interlaminar crack propagation of hybrid composites in interlaminar shear process was studied. Fig. S1 (b_2) showed that three cracks emerged in the TTCFC laminate at the same time. After point b_2, Fig. S1 (b_3) and Fig. S1 (b_4) showed there was only one crack in the laminate, respectively. For the CNTHC laminate, the first shear failure was accompanied by the occurrence of two cracks in Fig. S1 (c_2). Then, Fig. S1 (c_3) and Fig. S1 (c_4) displayed that each load drop was accompanied by one crack. When the ECNTHC appeared shear failure firstly, only one crack occurred, and the load dropped about 11%. As shown in Fig. S1(d), there was only one crack in each shear failure with slight load dropping.

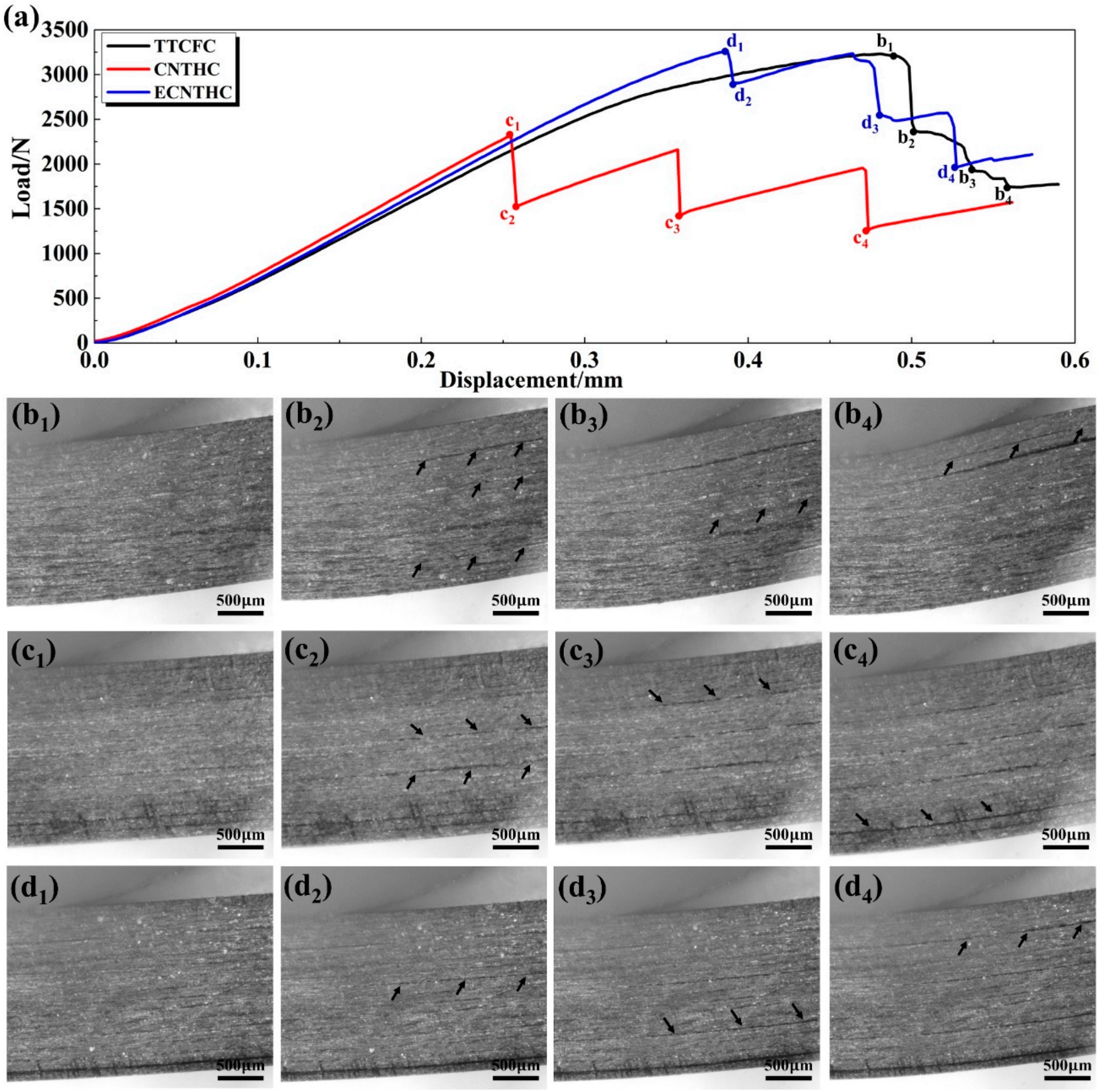

Figure S1. (a) Interlaminar shear load-displacement curves of TTCFC, CNTHC and ECNTHC. In-situ crack propagation images of (b) TTCFC (c) CNTHC and (d) ECNTHC.

Fig.S2 showed the first in-situ crack propagation images of the CNTHC $_{1/2}$, CNTHC $_{3/4}$, ECNTHC $_{1/2}$ and ECNTHC $_{3/4}$ laminates.

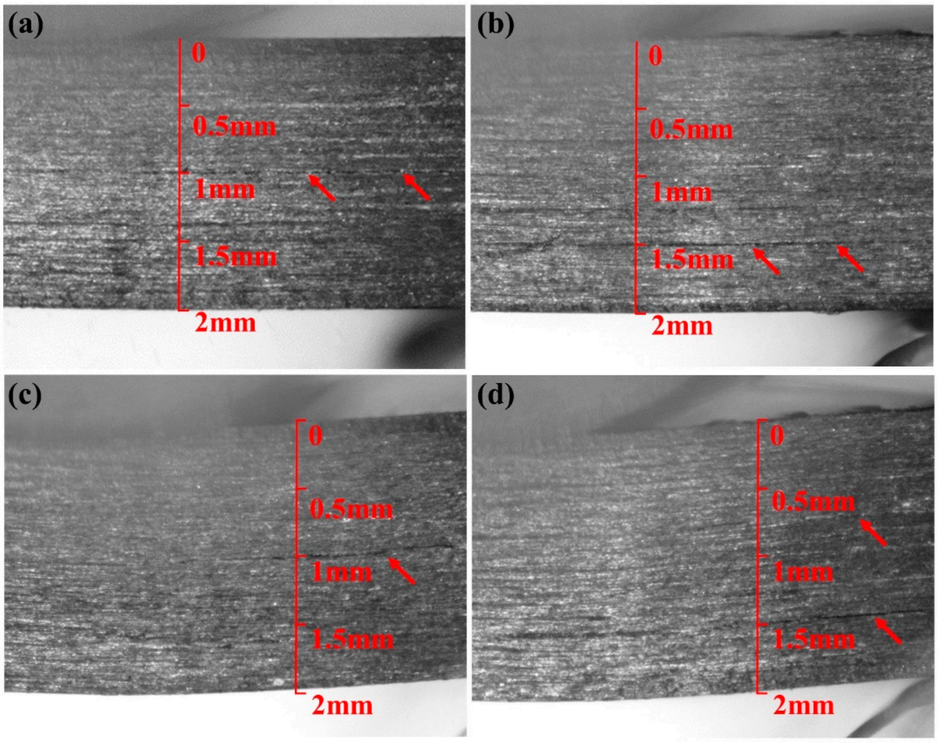

Figure S2. First in-situ crack propagation images of the (a) CNTHC $_{1/2}$ (b) CNTHC $_{3/4}$ (c) ECNTHC $_{1/2}$ and (d) ECNTHC $_{3/4}$.

Fig.S3 showed that the $\Delta R/R_0$ and $\frac{d(\Delta R/R_0)}{ds}$ of the TTCFC had irregular fluctuations, and $\frac{d(\Delta R/R_0)}{ds}$ had a maximum value in the non-crack propagation area, which could not be correlated with the sudden load change caused by crack propagation during the interlaminar shear. Therefore, for conventional CF composite, the interlayer delamination under shear stress could not be monitored by electric resistance changes.

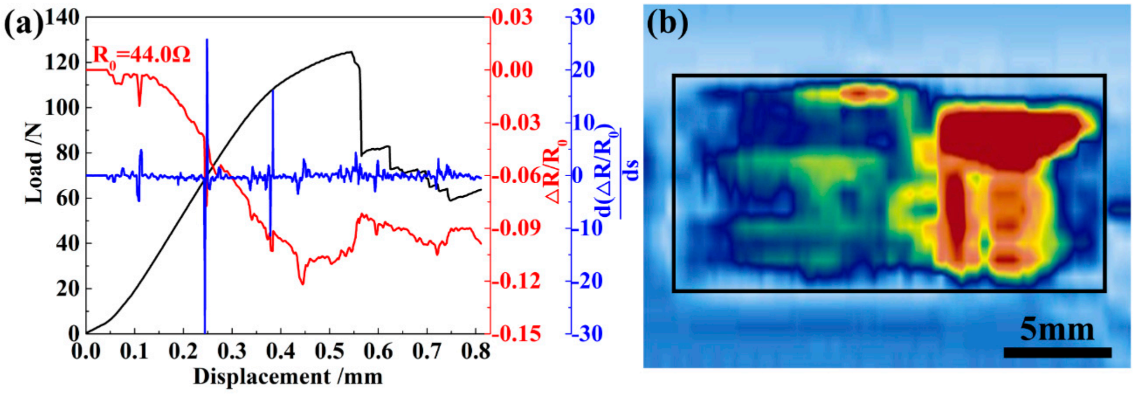

Figure S3. (a) Interlayer shear displacement-load-resistance change-resistance change derivative curve of TTCFC, (b) interlayer delamination of TTCFC by ultrasonic C scan.

Fig.S4 showed the in-situ crack propagation images of TTCFC and CNTHC laminates. Only the

indenter could be observed at the failure moment of the TTCFC (Fig. S4(a)). This indicated the TTCFC sample failed catastrophically and the crack propagation occurred instantaneously which the camera cannot capture. The inability of the thermoplastic tougheners to prevent the cracks from rapidly propagating through the thickness direction is the main reason for the low flexural strength of TTCFC. As for the CNTHC, Fig.S4(b) displayed a large number of interlaminar delamination failure morphologies which happened instantaneously. The CNT network structure had relatively higher mechanical properties than the thermoplastic toughener, which partly prevented cracks from propagating in the thickness direction. However, the weak nanoscale CNT/resin interface in CNTHC provided a path for cracks to spread through and along the interlayers, resulting in a large number of interlaminar delamination during bending failure. Energies were absorbed by the delamination, thereby, the bending performance of CNTHC was slightly improved.

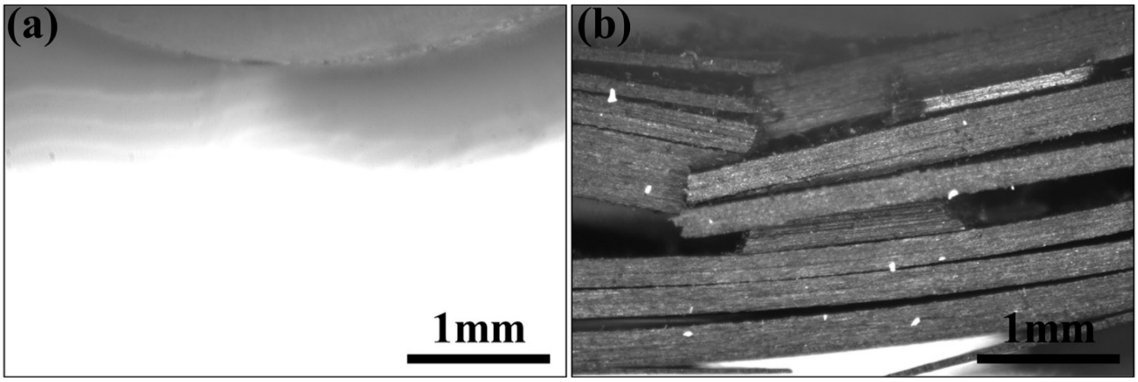

Figure S4. In situ crack propagation images of (a) TTCFC and (b) CNTHC laminates

The typical compressive failure morphologies of the TTCFC, CNTHC and ECNTHC were obtained by the 3D depth synthesis function of the 3D digital microscope. As shown in Fig. S5(a) and Fig. S5(c), the failure morphologies of the TTCFC and ECNTHC were similar. Due to the high interlaminar bonding strength, the different CF layers were coordinated to bear the load, then the kink band appeared and caused oblique failure. The fracture was complete and there were broken fiber slags on the fracture surface. Because of the weak nano CNT/resin interface, cracks first appeared and spread along the interlayer in the CNTHC under compressive load, which displayed a large number of delamination as shown in Fig.S5(b). There were clearly visible CNT networks on the delamination surface. The delamination caused the fiber layers to lose mutual support but also avoided mutual influence, presenting a single-layer or several-layer bending failure morphology together.

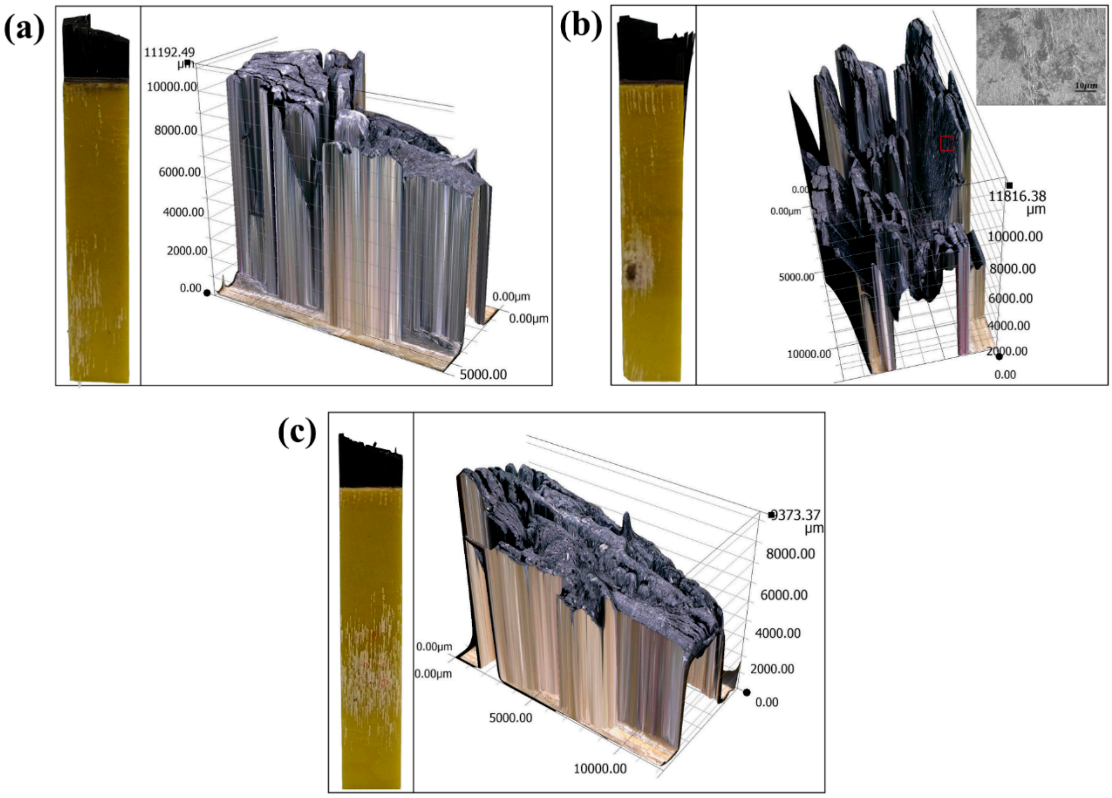

Figure S5. The Macroscopic 3D compressive fracture morphologies of (b) TTCFC, (c) CNTHC and (d) ECNTHC which obtained by 3D digital microscope.

The overlooking morphologies of the TTCFC, CNTHC and ECNTHC were obtained by using the 3D digital microscope, which were shown in Fig. S6(a)(b)(c) respectively. Fig.S6(g) displayed the outline height curves of the three macroscopic fractures along the width direction. The TTCFC and ECNTHC laminates all presented a kink band angle of 17~18° from the horizontal direction. The previous simulation and experiment results all recognized that the kink band angle was range from 17° to 20° [1], which also indicated that the laminates were damaged in a kink band failure mode. The partial enlarged morphologies of the TTCFC, CNTHC and ECNTHC laminates were shown in Fig.S6(d)(e)(f) respectively, which the height cloud images were employed to characterize the relative height. The red area was the high area, and the green area represented the low area in these figures. A gradual transition from red to green along the thickness direction was observed in Fig.S6 (d) and (f), indicating that the fractures of the TTCFC and ECNTHC also presented height gradient in the thickness direction. Considering the CNTHC laminate, the green color in Fig.S6(e) represented the interlaminar delamination, thus it was difficult to visually noticed the height variation along the thickness direction. As shown in Fig.S6(h), the height curves of the three magnified areas along thickness direction were

obtained. The curves of TTCFC and ECNTHC were still less fluctuating and a 21~22° kink band angle was displayed in the thickness direction. The curve of the CNTHC had a 20° angle despite large undulations.

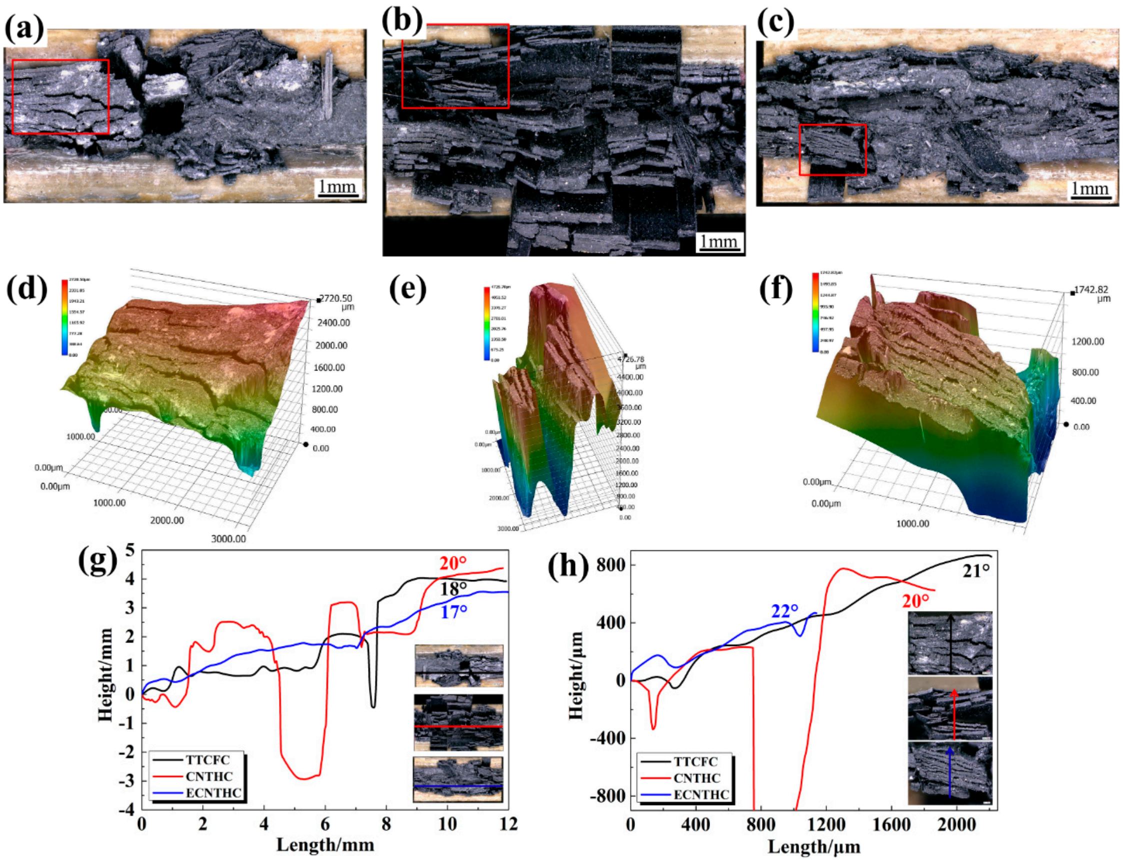

Figure S6. Top view of the compressive fracture morphologies of (a)TTCFC, (b) CNTHC and (c) ECNTHC. Local 3D fracture morphologies of (d) TTCFC, (e) CNTHC and (f) ECNTHC. Contour curves in the (g) width direction and (h) thickness direction of the three composites.

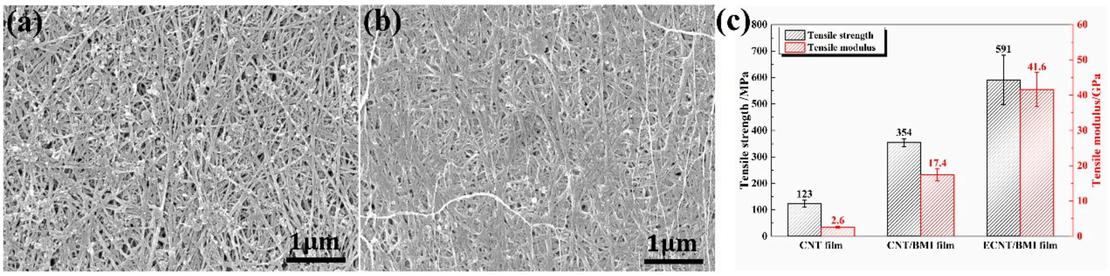

Figure S7. (a) SEM image of the pristine CNT network before chemical treatment, (b) SEM image of the CNT network after chemical treatment and (c) tensile properties of the pristine CNT film and its composite

Reference

[1] Gutkin R, Pinho ST, Robinson P, Curtis PT. On the transition from shear-driven fibre compressive

failure to fibre kinking in notched CFRP laminates under longitudinal compression. Composites Science and Technology. 2010;70(8):1223-31.
